# Metabolic Fingerprint of Chronic Obstructive Lung Diseases: A New Diagnostic Perspective

**DOI:** 10.3390/metabo9120290

**Published:** 2019-11-26

**Authors:** Dimitris Tsoukalas, Evangelia Sarandi, Maria Thanasoula, Anca Oana Docea, Gerasimos Tsilimidos, Daniela Calina, Aristides Tsatsakis

**Affiliations:** 1 Department of Clinical Pharmacy, University of Medicine and Pharmacy of Craiova, 200349 Craiova, Romania; calinadaniela@gmail.com; 2 Metabolomic Medicine Clinic, Health Clinics for Autoimmune and Chronic Diseases, 10674 Athens, Greece; research@metabolomicmedicine.com (E.S.); mariathanasoula84@gmail.com (M.T.); clinic@drtsoukalas.com (G.T.); 3 Laboratory of Toxicology and Forensic Sciences, Medical School, University of Crete, 71003 Heraklion, Greece; tsatsaka@uoc.gr; 4 Department of Toxicology, University of Medicine and Pharmacy of Craiova, 200349 Craiova, Romania; daoana00@gmail.com

**Keywords:** COLD, metabolomics, precision medicine, biomarkers

## Abstract

Chronic obstructive lung disease (COLD) is a group of airway diseases, previously known as emphysema and chronic bronchitis. The heterogeneity of COLD does not allow early diagnosis and leads to increased morbidity and mortality. The increasing number of COLD incidences stresses the need for precision medicine approaches that are specific to the patient. Metabolomics is an emerging technology that allows for the discrimination of metabolic changes in the cell as a result of environmental factors and specific genetic background. Thus, quantification of metabolites in human biofluids can provide insights into the metabolic state of the individual in real time and unravel the presence of, or predisposition to, a disease. In this article, the advantages of and potential barriers to putting metabolomics into clinical practice for COLD are discussed. Today, metabolomics is mostly lab-based, and research studies with novel COLD-specific biomarkers are continuously being published. Several obstacles in the research and the market field hamper the translation of these data into clinical practice. However, technological and computational advances will facilitate the clinical interpretation of data and provide healthcare professionals with the tools to prevent, diagnose, and treat COLD with precision in the coming decades.

## 1. Introduction

Chronic Obstructive Lung Disease (COLD) develops as a chronic and persistent inflammatory response to environmental stimuli. It is characterized by airflow limitation and affects the 10%–15% of the adult population globally, smoking being the most important risk factor for this disease [1]. COLD exacerbation is defined as an acute event caused by viral and bacterial infections with various germs such as *Haemophilus influenzae, Streptococcus pneumoniae* and sometimes *Staphylococcus aureus* that is difficult to treat [2,3,4]. More rare causes of exacerbation are: pneumothorax, pneumonia and parapneumonic pleurisy [5]. In these situations, the patient requires a complex treatment with antibiotics associated with glucocorticoids [6].

**Figure 1:**
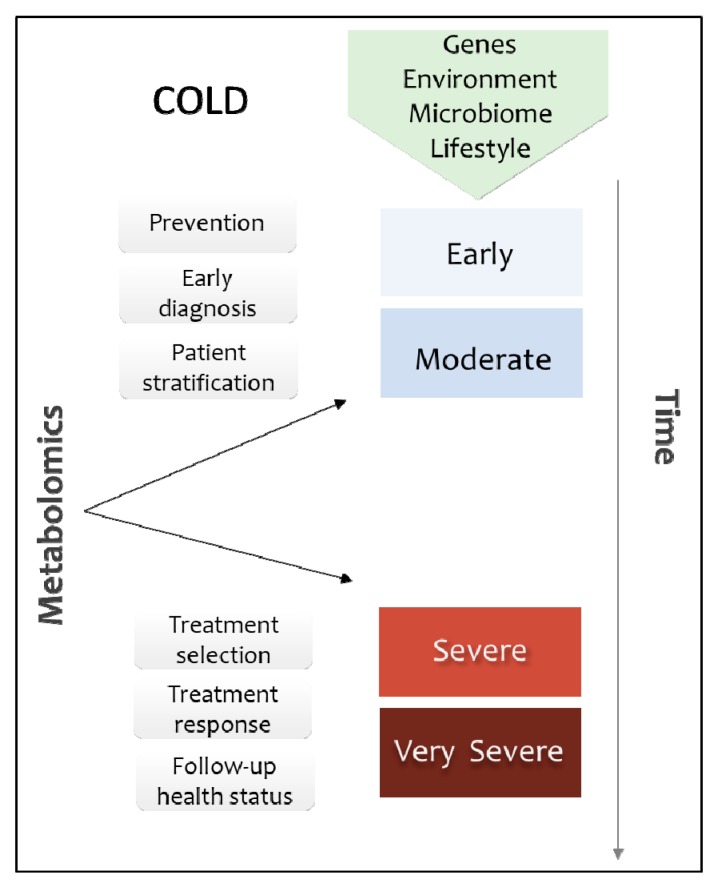
Metabolomics applications in COLD. Metabolites reflect the gene expression under the influence of environment, microbiome, and lifestyle, the combination of which is involved in the development of COLD. During the first stages of disease, metabolomic biomarkers can be used in prevention, early diagnosis, and patient stratification. In advanced stages the metabolic fingerprint can be used as a complementary tool for treatment selection and to monitor the treatment response and the overall health status of the patients in follow-up visits.

COLD is a very heterogeneous disease and therefore requires advanced and specialized methods for diagnosis [7]. Traditional methods for COLD diagnoses, such as forced expiratory volume in 1 second (FEV1) that measures the airflow limitation, are not suitable for all clinical cases, nor reliable for efficient prognosis, and are not able to clearly distinguish between different phenotypes and stages of the disease [8,9]. With the advent of precision medicine and patient-oriented approaches, metabolomics has gained great attention because it reflects the phenotype of the patient and can be used as a tool in early diagnosis, before the onset of symptoms, in treatment response monitoring, and to identify and target the metabolic profile of the disease [10] (Figure 1).

Several metabolomic studies in recent years have aimed to identify and validate specific biomarkers that would help in prognosis, as well as facilitating the early and precise diagnosis of the disease, considering the great heterogeneity of the pathogenesis of COLD [11]. The identification of specific biomarkers could also predict the response of COLD patients to different treatments depending on the cause, the stage, and the phenotype/subtype of the disease [12]. Many recent studies used measurement methods such as nuclear magnetic resonance (NMR), high performance liquid chromatography-mass spectrometry (HPLC-MS), and liquid chromatography-MS (LC-MS) for the detection of significant differences between metabolites that participate in various metabolic pathways in the plasma, serum, and urine of healthy individuals and COLD patients, COLD patients receiving different treatments, and patients with different phenotypes of COLD. The identification and validation of these biomarkers based on accurate measurements and validated standards are of crucial importance for the advancement of precision medicine and the unraveling of the pathogenesis in each COLD patient [13]. The aim of this article is to present findings from the biomarker discovery in COLD and discuss their potential application in clinical practice. Table 1 summarizes and compares the experimental design and the main findings of the metabolomic studies discussed in this article.

## 2. The Diagnostic Value of Metabolome in COLD

### 2.1. COLD Versus Healthy

There are two main analytical strategies used in metabolomics studies for the identification and evaluation of biomarkers, the untargeted and the targeted metabolomics analysis. The main differences between the two strategies are the level of sample preparation required, the number of the metabolites detected, and the level of the quantification of the metabolites. The selection of the appropriate strategy in each case is mostly based on these objectives [29]. More specifically, untargeted metabolomics is used to detect unknown metabolites and unexpected changes in metabolite concentrations, being able to measure a very large number of metabolites. They use a combination of multiple analytical methods in order to maximize the number of metabolites detected and increase the coverage of the metabolome. In untargeted metabolomics the sample preparation involves the extraction of the metabolites from the biological sample into a solvent that is suitable for the analytical method used. The analysis of the sample is performed with several analytical methods (such as NMR, LC-MS, or GC-MS), depending on the sample type and the objective of the analysis. During the analysis, the peak area of each metabolite that is recorded in the mass spectral analysis is selected, and statistical evaluation of the peak abundances is performed in order to define significant changes between the metabolic profiles of two or more groups under investigation [30]. The next step compares and assigns the signals obtained, with metabolite IDs existing in metabolomics databases. This approach provides a relative quantification of the metabolites, as the peaks obtained cannot be compared to calibration curves made with chemical standards, which is necessary for full quantification. Finally, the biological importance of each metabolite identified is determined by statistical analysis and biological interpretation. However, although great progress has been made in expanding metabolomics databases with new metabolites, a significant portion of signals detected in untargeted studies still cannot be identified due to the absence of their spectra in the databases. Identification of unknowns is generally accepted as the bottleneck of untargeted metabolomics.

On the other hand, targeted metabolomics aims to analyze specific metabolites and fully quantify their changes. It analyses a group of a small number of metabolites that are chemically characterized, predetermined, and involved in known metabolic pathways, providing their accurate identification and quantification. In targeted metabolomics, only the metabolites of interest are retained during sample preparation, while the rest of the biological species are removed. During the statistical analysis, itis determined how well the selected metabolites contribute to the separation of the groups under investigation, e.g., between the control and the phenotype of interest. As expected, data analysis and biological interpretation is simpler in studies that use targeted metabolomics compared to those using untargeted metabolomics. Moreover, targeted methods have a greater selectivity and sensitivity than untargeted methods, as they can be performed only if a standard curve of the metabolite is available. Finally, they perform full and accurate quantification of the selected metabolites by using internal and chemical standards for each of the metabolite in the study [31].

Most of the metabolomic data obtained the past few years on COLD were based on untargeted metabolomics studies aimed at the identification of unknown metabolites, serving as biomarkers. In particular, untargeted metabolomic studies sought to identify specific biomarkers for COLD by measuring differences in metabolites between healthy individuals and individuals with COLD. More specifically, several studies showed that aminoacid metabolism, lipid metabolism, energy metabolism, and oxidative stress pathways were disturbed in COLD patients compared to healthy controls. One of the biggest clinical studies, with respect to the number of individuals involved, included a control group of 66 healthy individuals and three groups of 70, 64 and 44 Global Initiative for Chronic Obstructive Lung Disease (GOLD) patients with different stages of COLD (stage II, stage III, and stage IV, respectively) [14]. In this study, NMR and LC-MS showed significant differences in metabolites involved in aminoacid metabolism including glutamine, phenylalanine, 3-methylhistidine, branched-chain amino acids (BCAAs) and glycine in the serum of COLD patients compared to the control group. Moreover, it showed differences in ketone bodies, lipoproteins, and creatine, which is involved in energy metabolism. Following this, a targeted metabolomic study on aminoacid metabolism showed increased glutamine, aspartate, and arginine in 30 COLD IV patients compared to 30 healthy individuals by LC-MS [29] confirming that pathways of aminoacid metabolism are often affected by COLD.

Similarly, in 2018, studies using HPLC-MS showed altered aminoacid metabolism, including decreased levels of the aminoacids arginine, proline, alanine, and phenylalanine. Moreover, the free carnitine to acylcarnitine ratio which is important for energy metabolism was decreased, while lysophosphatidylcholine, which is involved in lipid metabolism, was increased in the serum of COLD patients compared with healthy individuals [16,17]. Consistently, differences in acetate, ketone bodies, M-hydroxyphenylacetate, carnosine, pyruvate, phenylacetyglycine, 1-methylnicotinamide, creatinine, and lactate, many of them involved in energy metabolism, were found in the urine of 32 COLD patients compared with 21 healthy individuals, measured by NMR [18].

### 2.2. COLD Classification Based onGenetic and Environmental Factors

COLD is in many cases affected by environmental and genetic factors, a fact that makes the discovery of its pathogenesis even more complicated. According to the World Health Organization (WHO), several environmental factors cause COLD, such as air pollution, occupational dust, and chemicals, and frequent lower respiratory infections during childhood. However, tobacco smoke is the primary cause of COLD. Therefore, all the recent COLD studies assessing the effect of environmental factors on the metabolic profile of patients are focused on tobacco smoke. For example, 37 healthy smokers, 41 COLD-smokers, and 37 non-smokers participated in a study aimed at discovering serum metabolic markers associated with early-onset COLD [19]. It was found that out of 1181 distinct molecular ions detected in the sera of the subjects that participated in the study, 23 were differentially expressed in COLD-smokers versus healthy smokers. These biomarkers included fibrinogen peptide B, myoinositol, glycerophosphoinositol, fumarate, cysteinesulfonic acid, and creatinine that appeared to be increased in the COLD-smokers compared with the healthy smokers and some peptides with an undefined sequence that had chromatographic retention time consistent with fatty acids and lipids, involved in lipid metabolism. Furthermore, it was found in another study on healthy controls, healthy smokers, and smokers with COLD that fatty acids, sphingolipid pathways, and cyclic adenosine monophosphate (cAMP) signaling were disturbed in COLD-smokers compared with healthy smokers [20].

The only known genetic factor affecting COLD is ZZ-a1-antitrypsin deficiency which was associated with changes in the pyruvate metabolism mediators including acetoin, propionate, acetone, and propane-1,2 diol in the EBC from a group of ZZ-a1-antitrypsin-deficient COLD patients [28].

## 3. Matching the Right COLD Treatment to the Right Patient

In addition to the establishment of specific biomarkers necessary for prognosis and accurate diagnosis of COLD, the analysis of the effect of different treatments on metabolites of COLD patients is also of critical importance. COLD patients receiving standard therapy in combination with doxycycline treatment presented disturbed levels of formate, citrate, imidazole, L-arginine, lactate, and fatty acids compared with COLD patients receiving only the standard therapy measured in serum by NMR [12]. Moreover, 90 COLD patients after supplementation with anti-oxidant QTer (co-enzyme Q10 supplement) and creatine presented different levels of lysophosphatidylcholine, phosphatidylcholine and sphingomyelins in the plasma measured by LC-MS [21]. The authors concluded that the intervention resulted in improved respiratory function and body composition possibly linked to the changes in the metabolic profile. Finally, another intervention study analyzed the effect of endurance exercise for 8 weeks on COLD patients compared with healthy subjects [22]. According to this study, only the healthy group showed significant metabolic changes before and after training, including an increase in aminoacids, such as glutamine, tyrosine, alanine, valine, and isoleucine, as well as creatine, succinate, pyruvate, glucose, and lactate measured in their plasma. On the other hand, COLD patients showed only disturbed lactate levels in the plasma after exercise, supporting the idea that plasma metabolic profiling combined with different interventions can contribute to the phenotypic characterization of COLD patients and enhance our understanding of the involvement of muscle dysfunction.

Phenotyping of COLD has been challenging for both researchers and clinicians due to the heterogeneity of the disease. Thus, some studies have been focused on identifying different metabolic biomarkers between different COLD phenotypes and pathological profiles [9]. For example, a study comparing the plasma metabolites measured by LC-MS in 38 HIV-associated COLD patients with 38 healthy individuals, showed that kynurenine/tryptophan ratio, ceramide, and fatty acids were increased in the patients while diacylglycerol was decreased [23]. Moreover, serum and urine samples from stable COLD patients were compared with samples from acute respiratory failure patients caused by COLD exacerbation, pneumonia, and heart failure [24]. As measured by NMR, several metabolites including glutamine, alanine, proline, histidine, creatine, oxoglutarate, cis-aconitate, citrate, mannitol, niacinamide, nicotinamide, and furoylglycine exhibited different levels between stable COLD patients and patients with acute respiratory failure. These results suggest that discrimination between COLD phenotypes caused by different pathogenesis is possible due to different metabolites being affected in each case.

Another characteristic example of discriminating different COLD phenotypes based on metabolomics is two studies on COLD patients with phenotype E and phenotype M. COLD phenotypes E and M are two out of the three phenotypes that were identified according to the dominance of emphysema and the presence of bronchial wall thickening based on high-resolution computed tomography (HRCT). Phenotype E exhibits apparent emphysema without bronchial wall thickening, while phenotype M exhibits apparent emphysema with bronchial wall thickening. These studies showed disturbed levels of ADP, choline, glycine, threonine, proline, tyrosine, ornithine, L-alanine, L-valine, L-leucine, fructose, pyruvate, and isopropyl alcohol in the serum measured by NMR in both phenotypes [25,32]. L-threothine, creatine, citric acid, L-glutamine, maltose, ornithine, glutamic acid, asparagine, betaine, cyclopentane, and pyridoxine were affected only in type E COLD, while malonate, *N*-acetylcysteine, guanosine, and lipoprotein only in type M COLD. However, there is some inconsistency in the metabolic profiles between studies on phenotype E and M COLD. For example, guanosine appears perturbed in both phenotypes in one case [32] and only in type M in another case [25]. Moreover, pyruvic acid is affected in both type E and M COLD in one study and only in type E in another study. Such inconsistencies and discrepancies between metabolomic studies aimed at the identification of biomarkers for the same disease are commonly seen in heterogeneous and multifactorial diseases as the COLD.

## 4. Discussion

COLD is a chronic disease with various symptoms of the respiratory tract but also with a divergent metabolic fingerprint. A great number of COLD patients suffer from cachexia or metabolic syndrome, which has been strongly correlated to hospitalization and morbidity. The metabolic pressure in chronic diseases has been supported by several researchers stressing the need to find tools that can detect nutritional deficiencies, and metabolism disruptions that are related to the disease [33,34].

Untargeted metabolomic profiling of COLD patients has indicated several potential metabolite biomarkers that participate in aminoacid metabolism, lipid metabolism, and energy metabolism, as well as oxidative stress. From the pathways mentioned, the most commonly affected among the studies was the metabolism of aminoacids (Table 1). Levels of glutamine, phenylalanine, 3-methylhistidine, BCAAs, glycine, aspartate, arginine, alanine, cysteine, and ornithine were consistently affected in COLD patients compared to healthy controls. The most consistent metabolic pattern of amino acids in COLD includes reduced plasma levels of the BCAAs leucine, isoleucine, and valine, and a decreased muscle glutamate concentration. The reduction in glutamate status was linked to reduced muscle glutathione levels and enhanced glycolysis which is evident from the increase in plasma lactate during exercise in COLD patients [22,27]. Another COLD-associated metabolic pattern that is commonly observed includes perturbed plasma levels of alanine, tyrosine, glutamine, aspartic acid, lysine, proline, and ornithine in COLD patients compared with the control group (Table 1). These findings may result from the fact that patients with COLD have increased resting energy expenditure (REE) and are in a state of hypermetabolism, that is the increased consumption of calories per kilogram, to cover the increased effort for respiration [35,36,37]. This is further supported by the finding that low levels of BCAA/AAA (aromatic amino acids) and increased REE were significantly correlated with the percentage of ideal body weight, percentage of arm-muscle circumference, and % FEV1, especially in underweight COLD patients [26]. The authors suggested that disturbed aminoacid metabolism is a characteristic of underweight COLD patients and may be linked to disease severity and reduced respiratory muscle efficiency.

However, as already mentioned, there is some variability and inconsistency between the results of different metabolomic studies on COLD that hampers the validation of biomarkers. This could be explained due to different measurement techniques and standards used in each study, different sample preparation, handling and processing conditions, different quality control samples and internal standards, and different number of replicates used in each study that are rarely mentioned [12], different characteristics of the subjects involved, including age, sex, physical activity, nutritional status, and exposure to different environmental conditions, and very importantly the heterogeneity of COLD with respect to the lung function and its pathogenesis [11,38]. In particular, the quality controls and internal standards used among studies varied significantly, with some of the most commonly used being, either a mix of known metabolites that were placed randomly in the MS run, or a mixture of the sample with isotopically labelled internal standards of diagnostic metabolites, or a pooled extract from all serum samples, for example, that was stored in single-thaw aliquots and used to correct for potential day-to-day and batch-to-batch LC/MS data drift [17,19,23,24]. Intra-individual and inter-individual variability is a major limitation of the studies that aim to find the metabolic signature of COLD. Appropriate selection of patients, detailed medical, nutritional and lifestyle history, and dosing treatment history are crucial in these studies. However, the collection and processing of this large amount of data can be challenging for large studies. Thus electronic health records (EHR) can be a very helpful tool of precision medicine [13]. In addition, the clinical interpretability of results is affected by the sampling site. As has been discussed elsewhere, peripheral blood which the most commonly used sample site, may not be representative for some diseases [39]. In COLD, analyzing samples from different sites, e.g., bronchoalveolar lavage fluid (BALF), exhaled breath condensate (EBC) and plasma can be very useful for the thorough characterization of the pathologic profile of each patient [40]. Breath analysis has already provided promising results for infections and lung cancer diagnosis setting the grounds for other diseases, including COLD [41].

The major problem of modern medical science, according to the medical doctor Seth J. Baum, lies on the lack of communication between clinicians and researchers leading to the poor interpretability of human studies [42]. In the case of metabolomics, most data are obtained from untargeted analysis in patients compared to non-patients aiming to find “metabolic hits” that could be used to discriminate the two groups. However, in a study by Kilk K and his team, it was shown that COLD patients were discriminated from the control group only when results from the targeted analysis were assessed and not the whole metabolic profile [16]. In particular, they integrated untargeted metabolomic data from two different sample specimens (peripheral blood and EBC), demographic and clinical characteristics, and targeted metabolomic analysis of peripheral blood. Combination of clinical/demographic and untargeted metabolomic data was used in order to find associations between clinical/demographic and metabolic parameters that would allow an attempt at de novo phenotyping of COLD. The authors discuss that in addition to the targeted metabolic profile, clinical parameters could also discriminate COLD patients from healthy participants which is reasonable considering that the COLD diagnosis is based on these clinical parameters.

**Figure 2:**
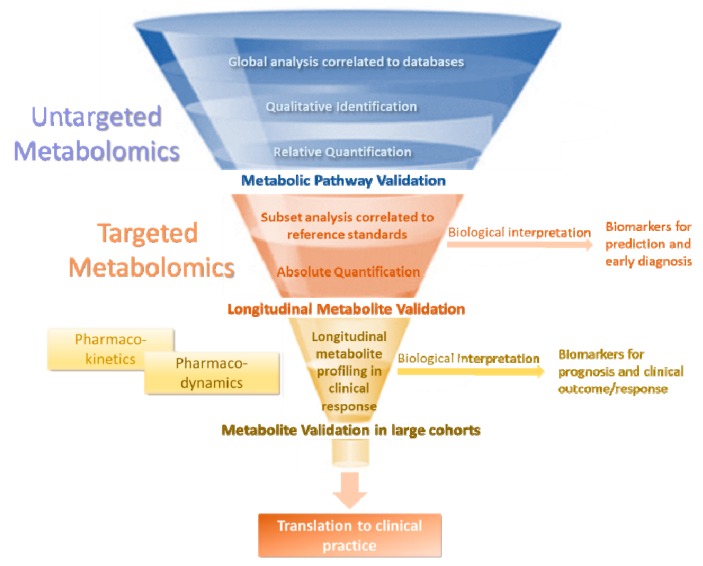
The steps of the discovery and validation of metabolomic biomarkers. Untargeted metabolomics identifies candidate biomarkers using global databases which are introduced to the validation stage. Initial validation is achieved with targeted metabolomics which is a quantitative analysis of well-defined and pre-selected subsets of metabolites that leads to the generation of biomarkers for prediction, prevention, and early diagnosis of a disease. Biomarkers for prognosis and clinical outcome can be generated by integrating the metabolites from targeted metabolomics in longitudinal studies. Large cohorts using these biomarkers can lead to further validation and application to clinical practice.

As has been discussed elsewhere, biomarker discovery and validation should follow a multistep approach using untargeted metabolomics for the initial screening followed by quantitative analysis through targeted metabolomics and biological interpretation of the results [39] (Figure 2). Specifically, untargeted metabolomics focuses on the global detection of small molecules in a sample and correlates them to known libraries and databases. It is a pre-validation phase that is often performed in small cohorts focused on the qualitative identification and relative quantification of the metabolites detected, in order to generate a hypothesis which should be further validated by targeted metabolomics. Following the pre-validation phase, targeted metabolomics focuses on the validation of an existing hypothesis by measuring well-defined subsets of metabolites through their correlation with reference standards [43,44]. It offers the opportunity of absolute quantification of the metabolites measured and defines the metabolic pathway involved in the pathogenesis of a disease. This leads to the assessment of biomarkers for prediction, prevention, and early diagnosis of a disease [45]. As a next step, the longitudinal dynamic profiling of the identified biomarkers is crucial for their longitudinal validation based on pharmacodynamics and pharmacokinetics. The biological interpretation of this data allows the establishment of dynamic biomarkers that could predict and describe the inter-individual variation in disease progression, treatment response, and clinical outcome. Finally, validation studies in larger patient cohorts that assess the robustness of the discovered biomarkers are the last validation step allowing their application in individualized clinical practice.

Improvements and major future steps must be taken in the field of metabolomics in general, regarding more reliable and precise metabolite identification, data analysis and interpretation of the biological relevance of the biomarkers identified. As already mentioned, metabolite identification in untargeted studies remains the main bottleneck in data analysis and interpretation [46]. In contrast to genomics, there is a lack of metabolite databases with enough depth and breadth covering entire metabolomes and including all the molecular diversity that is available. There are two main types of resource available in the field at the moment, databases of measurements and genome-based metabolic reconstruction databases, but these do not fulfill the needs of metabolomics. To achieve efficient metabolite identification, coordinated effort across research groups and countries worldwide is required, with many resources and developments employed. This concerted effort is crucial to enrich databases with long-term supported registries of tools and standards that will help researchers decide which well-tested tools and standards to use for each purpose [47]. However, there is not only a lack of metabolites’ profiles in the metabolomics databases, but there are also different confidence levels provided by databases based on the structural identification of the metabolites [48]. To achieve the highest confidence level, there should be authentic chemical standards available, that can be matched to the data from the biological samples analyzed. However, this process is hampered by the lack of availability of authentic chemical standards for many metabolites, a problem that can be solved with the contribution of bioinformatics and chemoinformatics. Finally, interpretation of the biological relevance of measured metabolites will be facilitated by the mapping of their chemical structures. This can be achieved by using a combined multi-omics data integration approach, which could eliminate the differences between different databases, as one may focus on the biological role of the metabolite, while another on the metabolites’ chemical structure and properties.

The goal of precision medicine is to provide tailor made treatment for all, considering the inter-individual variation in treatment response. Pharmacometabolomics is an emerging and rapidly evolving field regarding the identification of metabolic biomarkers or networks that will facilitate the treatment selection and appropriate dose. It was first introduced by Clayton et al., who developed a predictive model based on the metabolic changes of rats after treatment compared to the pre-treatment profile [49]. Today the scientific community focuses on the identification of metabolic biomarkers of disease progression and treatment response which requires longitudinal studies able to capture the dynamic metabolites combining pharmacokinetic and pharmacodynamics [27].

There is accumulating evidence which suggests that nutritional deficiency plays a key role in COLD patients which partially explains the metabolic imbalances that are observed in the metabolomic profile of these patients [50]. Although it remains to be unraveled to which extent these metabolic alterations are the cause or a symptom of the disease, nutritional interventions have been suggested to be beneficial. Intake of omega-3 polyunsaturated fatty acids has been shown to exert anti-inflammatory effects in chronic respiratory conditions such as asthma and COLD [51]. For example, in a double blind 6 months Randomized Control Trial (RCT) on asthma we showed that fatty fish intake significantly reduced the levels of the bronchial inflammation marker, eNO, which was correlated to the serum increase of docosahexaenoic acid (DHA), eicosapentaenoic acid (EPA), and omega6/omega3 ratio reduction after the intervention [52]. Furthermore, the immune-modulatory effect of Vitamin D has been shown to be important for the improvement of muscle weakness and exacerbation events in COLD patients [53,54]. Therefore, metabolomics could be used as an assessment tool of the nutritional needs of patients with COLD for interventional studies on the efficacy of nutrients on disease prevention and treatment.

The outstanding advantage of metabolomics is that the clinical doctor will be able to provide a personalized diagnosis and treatment according to the patient’s metabolic profile and not the symptomatology [38]. There is growing evidence that chronic malnutrition is a common denominator in many chronic diseases, including COLD, and our aim is to detect and target the metabolic pathways that have been affected [17,55]. We intend to continue our research in this direction and determine the metabolic fingerprint of COLD and other chronic diseases and provide evidence on the beneficial role of nutritional intervention to the metabolic background of these diseases.

## 5. Conclusions

Overall, even if great progress has been made in the field of metabolomics for COLD diagnosis and treatment, and a big range of metabolites are suggested as potential biomarkers for COLD most of them are still not validated. Common metabolic patterns of COLD patients include changes in the aminoacid metabolism and the ratios of certain aminoacid groups such as the BCAA/AAA ratio. These metabolic biomarkers have been associated with clinical symptoms, such as weight loss and reduced % FEV1, contributing to the selection of the right specialized treatment for each clinical case. However, large and longitudinal studies with a thorough analysis of the demographic variables of patients and the metabolic profiles will facilitate the validation of these biomarkers. The majority of the metabolomic studies were research-based, performing untargeted analysis of COLD patients and ending up with a large amount of metabolomic data that was difficult for scientists and clinicians to use. Tools of precision medicine, including HER and computational analysis, will help the management and analysis of data, and pharmacometabolomics will bring precision medicine closer to clinical practice.

## Figures and Tables

**Table 1:** Summary of COPD-associated metabolomic studies, including participant characteristics, inclusion/exclusion criteria for participant selection, sample type analyzed, analytical method used, metabolomic profiling results, and parameters and confounders used for analysis.

Study	Subjects	Criteria	Sample	Method	Metabolites	Confounders
Ubhi et al., [14]	Control: *n* = 66: non-smokers: *n* = 15 (8M/7F), Age: 61, BMI:27.7 smokers: *n* = 53, (34M/19F), Age: 57, BMI: 28.6 Patients: *n* =163 GOLD II: *n* =69, (46M/23F), Age: 65, BMI:27.9 GOLD III: *n* = 63 (43M/20F), Age: 64, BMI: 26.9 GOLD IV: *n* = 31, (18M/13F), Age: 63, BMI: 25.8	Controls and COLD patients were matched for sex, age, and smoking history	Serum	Untargeted NMR/LC-MS/MS	glutamine, phenylalanine, creatine, glycine, methionine, glycerol, monoglyceride, trimethylamine BCAA degradation: isobutyrate, 3-hydroxyisobutyrate, isoleucine, leucine, valine Lipid metabolism: HDL,LDL/VLDL, monoglyceride, glycerol Ketone bodies: acetoacetate, ascorbate, 3-hydroxybutyrate	Analysis based on GOLD stage, cachexia, emphysema, diabetes, patient location, age, sex, and comorbidities
Ubhi et al., [15]	Control: *n* =30 (30 M), Age:57, BMI: 29.9 Patients: *n* = 30 GOLD IV (30 M), Age:65, BMI: 26.2	Inclusion Control: aged 40–75, current or ex-smokers with >10 pack–year history, postbronchodilator FEV1 < 80% of predicted normal and FEV1/FVC ratio < 0.7. Patients: smoking (≥ 10 pack–years) and non-smoking (<1 pack–year). Control subjects: aged 40–75 years with normal lung function (post-bronchodilator FEV1>85% predicted and FEV1/FVC >0.7).	Serum	Targeted LC-MS/MS	glutamine, arginine, aspartate, aminoadipic acid, proline, leucine, valine, isoleucine, g-aminobutyric acid, a-aminobutyric acid, 4-hydroxyproline	Aminoacids profile analysis based on weight, BMI, age, and sex
Kilk et al., 2018 [16]	Control: *n* =21 (9M/12F), Age: 37, BMI: 24 Patients: *n* =25 (25M), Age: 67, BMI: 26	De novo phenotyping according to characteristics, medication, and co-morbidities pulmonary function	Blood/ EBC	Untargeted HPLC-MS	carnitine, glutamine, histidine, lysine, kynurenine, putrescine, lysoPC	Analysis based on clinical parameters and metabolomics
Novotna et al., 2018 [17]	Control: *n* = 10 (5M/5F), Age: 61.5, BMI: 25.3 Patients: *n* = 10 (5M/5F), Age:55, BMI: 27.1	Inclusion: Patients: non-smokers or ex-smokers >6 months, patients without acute exposition to carbon monoxide, COLD patients with post-bronchodilator values FEV1 < 60%. Exclusion: current smokers or ex-smokers <6 months, with a known metabolic disease or kidney disease, or presence of coronary artery disease.	Blood	Untargeted HPLC-MS/MS	carnitine, phenylalanine, tyrosine, carnitine/ acycarnitine, valine, methionine, glycine, leucine, isoleucine,	Analysis of different metabolic profiles based on age, sex, and BMI
Wang et al., 2013 [18]	Patients Phenotype E: *n* =22 (20M/2F), Age: 73.64, BMI: 21.21 Phenotype M: *n* =28 (25M/3F), Age:70.18, BMI: 19.65	Exclusion: respiratory tract infection, exacerbation of an airway disease in the previous 3 weeks, associated respiratory diseases, serious cardiovascular disease, cancer, cognitive impairment, immunodeficiency, or unable to complete protocol+D10	Serum	Untargeted NMR	ADP, guanosine, tyrosine, uridine, maltose, sucrose, L-threonine, D-glucose, glycine, proline, betaine, choline, malonate, L-lysine, creatine, asparagine, aspartate, succinate, pyruvic acid, acetone, ornithine, L-alanine, lactate, isopropyl alcohol, L-valine, leucine	No information provided
Chen et al., 2015 [19]	Control: Non-smokers: *n* =37 (19M/18F), Age: 39.5, BMI: 26.6 Smokers: *n* =40 (35M/5F), Age: 41.8, BMI: 26.9 Patients Smokers: *n* =41 (38M/3F), Age: 53.2, BMI: 25.6	Exclusion: non-smokers with no prior exposure to cigarette smoking and no detectable nicotine metabolites	Serum	Untargeted LC-MS	cotinine, 3-hydroxycotinin, Quinic acid, glycochenodeoxycholic acid 3-glucuronide, cysteinsulfonic acid, glycerophosphoinositol, phosphatidylinositol, creatinine, myoinositol, fibrinogen peptide B, hydrophobic unknowns	Analysis based on smoking status and clinical lung function parameters
Naz et al., 2017 [20]	Control: Non-smokers: *n* =38 (20M/18F), Age M: 62, Age F: 55.5, BMI M: 25.6, BMI F: 26.5 Smokers: *n* =40 (20M/20F), Age M: 52.5, Age F: 54, BMI M: 25, BM1 F: 24.2 Patients: Smokers: *n* =27 (15M/12F), Age M: 61, Age F: 59, BM1 M: 24.2, BMI F: 23.5 Ex-smokers: *n* =11 (5 M/6 F), Age M: 64, Age F: 58, BMI M: 29.1, BMI F: 27.6	Inclusion: Patients: no allergy or asthma history, no use of inhaled or oral corticosteroids, and no exacerbations for at least 3 months prior to study COLD patients and smokers matched for smoking history and current smoking habits	Serum	Untargeted LC-MS	Both sexes: citrate cycle, glycerophospholipid metabolism, pyruvate metabolism Sex-enhanced - female COLD: Fatty acid biosynthesis, sphingolipid metabolism Sex-enhanced - male COLD: cAMP signaling pathway, retrograde endocannabinoid signaling, tryptophan metabolism	Sex-specific metabolomic analysis
De Benedetto et al., 2018 [21]	Patients: Active Coenzyme Q10(QTer) *n* =45(34M/11F), Age: 73, BMI: 31.2 Placebo: *n* =45 (34M/11F), Age: 73, BMI: 29.6	Inclusion: clinically stable, no COLD exacerbation or hospitalization 4 weeks prior to enrolment, or receiving bronchodilator treatment Exclusion: mechanical ventilation, uncontrolled diabetes mellitus, severe heart, renal or hepatic failure and current or pre-existing malignant disease within the 3 years, persistent infections, chronic oral steroid or immunosuppressive therapy, or inability to complete tests and use of statins or amino acid supplements	Plasma	Untargeted LC-MS	lysophosphatidyicholine, phosphatidylcholine, sphingomyelins	No information provided
Rodríguez et al., 2012 [22]	Controls: *n* =12 (10M/2F), Age: 65, BMI: 26 Patients: *n* =18 (17M/1F), Age: 68, BMI: 24	Inclusion: no COLD exacerbations, no oral steroid treatment in the previous 4 months, all on bronchodilators and inhaled corticosteroids, and no major co-morbidities	Plasma	Untargeted NMR	glutamine, tyrosine, alanine, valine, isoleucine, creatine, creatinine, citrate, glucose, lactate, succinate, pyruvate	No information provided
Hodgson et al., 2017 [23]	HIV(+)COLD(+): *n* =38 (27M/11F), Age: 38.97 HIV(+)COLD(-): *n*=40(29M/11F) Age: 38.93 HIV(-)COLD(+): *n* =20 (18M/2F) Age: 48.18 HIV(-) COLD(-): *n* =17 (15M/2F), Age: 55.91	Inclusion HIV-positive controls: normal lung function, matched on age, sex, region, and smoking status HIV-negative controls: from the COPDGene study, matched on lung function, age, sex, and race	Plasma	Untargeted LC-MS/MS	ceramide, fatty acids, diacyglycerol, kynurenine/tryptophan ratio	HIV-associated metabolomic analysis
Fortis et al., 2017 [24]	Stable COLD: *n* =15 (6M/9F), Age: 68, BMI: 29.25 AECOLD: *n* =12 (4M/8F), Age: 73.1, BMI: 28.8 CHF: *n* =8 (3M/5F), Age: 78.5, BMI: 29.1 PNA: *n* =9 (6M/ 3F), Age: 65.7, BMI: 29.8	Inclusion Stable COLD: COLD diagnosis, smoking history, FEV1/FVC<lower limit of normal, FEV1%predicted<60% on stable respiratory condition AECOLD:COLD exacerbation, >40 years old, smoking history>20 pack-years with COLD, or COLD confirmed with PFTs CHF: Acute decompensate (systolic or diastolic) heart failure, defined as change in baseline dyspnea with evidence of fluid overload, elevated natriuretic peptides, or known history of chronic systolic or diastolic heart failure PNA: Pneumonia, defined as new infiltrate on admission CXR and symptoms consistent with pneumonia: malaise, sputum production, fever (T > 38.0°C), and crackles in auscultation of lung Exclusion: History of both COLD and heart failure, admitted with acute respiratory failure due to more than one reason (e.g., COLD and CHF, COLD and PNA, or CHF and PNA), previously diagnosed with bronchial asthma, bronchiectasis, bronchiolitis related to systemic pathology, cystic fibrosis, obstructive sleep apnea, or upper airway obstruction	Serum/ urine	Untargeted NMR	glycine, glutamine, alanine, proline, glutamate, mannitol, citrate, histidine, formate, creatine phosphate	Metabolomic analysis based on different clinical characteristics of COLD patients
Tan et al., 2018 [25]	Control: *n* =24 (14M/10F), Age: 61.5, BMI: 20.1 Patients: Phenotype E: *n* =20 (9M/11F), Age: 60.6, BMI: 19.1 Phenotype M: *n* =22 (14M/8F), Age: 62, BMI: 19.8	Exclusion: other diseases and use of other medication	Serum	Untargeted NMR	Phenotype E vs. control: lactate, fructose, glycine, creatine, asparagine, citric acid, pyruvic acid, pyruvate, proline, acetone, L-glutamine, L-proline, ornithine, lipid CH2CH2CO, 2-hydroxyisobutyrate, threonine, isopropyl alcohol, pyridoxine, maltose, L-threonine, L-valine, glutamic acid, beta-alanine, cyclopentane, 2-aminoisobutyric acid Phenotype M vs. control: fructose, glycine, pyruvic acid, pyruvate, proline, acetone, L-proline, ornithine, lipid CH2CH2CO, threonine, isopropyl alcohol, guanosine, betaine, N-Acetyl-Cysteine(NAC),lipoprotein, L-alanine Phenotype E vs. Phenotype M: L-glutamine, L-alanine	Analysis based on lung function, serum samples, medical history, age, sex, smoking, physical examination, and scores of COLD assessment test
Yoneda et al., 2001 [26]	Controls: *n* =30 (29M/1F), Age: 64 Patients: *n* =30 (29M/1F) Age: 64	Exclusion: other causes of weight loss (diabetes, endocrine disorders, malabsorption syndrome, neoplastic, infectious or liver diseases). Inclusion: Patients: receiving anticholinergic drugs, no requirement of supplemental oxygen, and no treatment with glucocorticoids or theophylline. Controls and patients matched for smoking habits	Plasma	Untargeted LC-MS	threonine, valine, leucine, isoleucine, methionine, phenylalanine, lysine, taurine, aspartic acid, glutamic acid, glutamine, serine, proline, glycine, alanine, tyrosine, ornithine, cysteine, histidine, arginine, BCAA, AAA, BCAA/AAA	Aminoacid analysis and BCAA/AAA ratio
Singh et al., 2017 [12]	COLD patients: Standard therapy: *n* =20 (20 M) Age: 64.2, BMI: 23.2 Standard+Doxy: *n* =30 (30M) Age: 67, BMI: 22.7	Exclusion: significant cardiac and other co-morbidities, history of exacerbations in the preceding 6 weeks, and history of doxycycline intolerance or co-existing pulmonary condition affecting the assessment or intervention for COLD	Serum	Untargeted NMR	formate, citrate, imidazole, lactate, L-arginine, fatty acid	No information provided
Engelen et al., 2000 [27]	Control: Physically inactive: *n*= 15(10M/5F), Age: 67 Physically active: *n* =7(7M), Age: 63 Patients: EMPH+ (with macroscopic emphysema): *n* = 12 (10M/2F), Age: 64 EMPH- (without macroscopic emphysema): *n* =15 (11M/4F), Age: 64	Inclusion: Patients: chronic airflow limitation (FEV1 < 70%),irreversible obstructive airway disease (<10% improvement of predicted baseline FEV1 after inhalation of b2antagonist), in clinically stable condition and without respiratory tract infection or exacerbation of their disease for at least 4 weeks before the study Exclusion: malignancy, cardiac failure, distal arteriopathy, recent surgery, severe endocrine, hepatic, or renal disorder and use of anticoagulant medication	Muscle biopsy/serum	Untargeted HPLC	glutamate, glycogen, glucose, pyruvate, lactate, lactate/pyruvate	Analysis of physical activity-dependent metabolic profiles
Airoldi et al., 2016 [28]	Controls: *n*= 11(4M/7F), Age: 55.27 Patients: *n* =11 (8M/3F), Age: 53	Inclusion: protease inhibitor genotype ZZ-α1-antitrypsin deficient(PiZZ-AATD)patients with pulmonary emphysema recruited from the Department of Pulmonology of Leiden University Medical Center, The Netherlands Control group with non-smoking healthy volunteers, with normal spirometry results and no significant history of respiratory diseases	EBC	Untargeted NMR	acetate, 2,3-butanediol, propionic acid, lactate, butyrate, acetone, benzoate, fatty acid, formate, propylen glycol, alanine, ethanol, acetoion, isopropanol	No information provided

Abbreviations: GOLD: Global Initiative for Chronic Obstructive Lung Disease; BCAA: branch chain amino acids; AAA: aromatic amino acids; FEV1: forced expiratory volume in 1 second; FVC: forced vital capacity; CXR: chest X-ray; COLDGene: Genetic Epidemiology of Chronic Obstructive Pulmonary Disease; Doxy: doxycycline; PFTs: pulmonary function tests; EBC: exhaled breath condensate; NMR: nuclear magnetic resonance; LC-MS: liquid chromatography-mass spectrometry; HPLC-MS: high performance liquid chromatography-mass spectrometry.
